# Geriatric Trauma Patients With Cervical Spine Fractures due to Ground Level Fall: Five Years Experience in a Level One Trauma Center

**DOI:** 10.4021/jocmr1227w

**Published:** 2013-02-25

**Authors:** Hao Wang, Marco Coppola, Richard D. Robinson, James T. Scribner, Veer Vithalani, Carrie E. de Moor, Raj R. Gandhi, Mandy Burton, Kathleen A. Delaney

**Affiliations:** a Department of Emergency Medicine, JPS Health Network, 1500 S. Main St. Fort Worth, TX 76104, USA; b Department of Trauma, JPS Health Network, 1500 S. Main St. Fort Worth, TX 76104, USA

**Keywords:** Geriatric, Trauma, Ground level fall, Cervical spine fracture, Alcohol

## Abstract

**Background:**

It has been found that significantly different clinical outcomes occur in trauma patients with different mechanisms of injury. Ground level falls (GLF) are usually considered “minor trauma” with less injury occurred in general. However, it is not uncommon that geriatric trauma patients sustain cervical spine (C-spine) fractures with other associated injuries due to GLF or less. The aim of this study is to determine the injury patterns and the roles of clinical risk factors in these geriatric trauma patients.

**Methods:**

Data were reviewed from the institutional trauma registry of our local level 1 trauma center. All patients had sustained C-spine fracture(s). Basic clinical characteristics, the distribution of C-spine fracture(s), and mechanism of injury in geriatric patients (65 years or older) were compared with those less than 65 years old. Furthermore, different clinical variables including age, gender, Glasgow coma scale (GCS), blood alcohol level, and co-existing injuries were analyzed by multivariate logistic regression in geriatric trauma patients due to GLF and internally validated by random bootstrapping technique.

**Results:**

From 2006 - 2010, a total of 12,805 trauma patients were included in trauma registry, of which 726 (5.67%) had sustained C-spine fracture(s). Among all C-spine fracture patients, 19.15% (139/726) were geriatric patients. Of these geriatric patients 27.34% (38/139) and 53.96% (75/139) had C1 and C2 fractures compared with 13.63% (80/587) and 21.98% (129/587) in young trauma patients (P < 0.001). Of geriatric trauma patients 13.67% (19/139) and 18.71% (26/139) had C6 and C7 fractures compared with 32.03% (188/587) and 41.40% (243/587) in younger ones separately (P < 0.001). Furthermore, 53.96% (75/139) geriatric patients had sustained C-spine fractures due to GLF with more upper C-spine fractures (C1 and C2). Only 3.2% of those had positive blood alcohol levels compared with 52.9% of younger patients (P < 0.001). In addition, 6.34% of geriatric patients due to GLF had intracranial pathology (ICP) which was one of the most common co-injuries with C-spine fractures. Logistic regression analysis showed the adjusted odds ratios of 1.17 (age) and 91.57 (male) in geriatric GLF patients to predict this co-injury pattern of C-spine fracture and ICP.

**Conclusion:**

Geriatric patients tend to sustain more upper C-spine fractures than non-geriatric patients regardless of the mechanisms. GLF or less not only can cause isolated C-spines fracture(s) but also lead to other significant injuries with ICP as the most common one in geriatric patients. Advanced age and male are two risk factors that can predict this co-injury pattern. In addition, it seems that alcohol plays no role in the cause of GLF in geriatric trauma patients.

## Introduction

Trauma is one of the common causes of Emergency Department (ED) visit in US [1, 2]. The clinical outcomes of the trauma patients vary depending on different trauma mechanism(s), injury patterns, and populations [3, 4]. In recent years, with the increase of geriatric population, the number of geriatric trauma patients has increased approximately 3-5% annually [5-7]. Results of previous studies on comparing geriatric trauma patients and non-geriatric group with the same injury mechanism showed that geriatric patients tended to sustain severe injuries, have worsening clinical outcomes, and relatively be underestimated at triage [8, 9]. This could partially attribute to the unique pathophysiological changes in geriatric population including degenerated joint changes, lesser mobility of vertebral spines, less muscle or ligament support, and decreased multi-organ functional reservoir [10-12]. Therefore, it is necessary to consider geriatric trauma patients as a special trauma population.

In general, a ground level fall (GLF), or a fall from a lower-height-than a GLF, is considered minor trauma and patients sustain less traumatic injury. However, in geriatric patients, severe injuries due to a GLF are relatively common [13]. Intracranial head injury, contusions, lacerations, and fractures all occur frequently, and C-spine fractures and intracranial pathology are most commonly seen in the geriatric trauma patients with GLF [14].

Understanding of an injury event, the mechanical forces involved, the significant biomechanical changes, and its related clinical variables in geriatric trauma patients will be important to predict the likelihood and severity of specific injury patterns. This will assist Emergency Physician in the appropriate management and disposition of these patients. At present, it is still uncertain whether any associated high risk injuries could be co-existed in geriatric trauma patient due to GLF and whether any other independent risk factors that could attribute to these injuries. Therefore, the aim of this study is to determine the injury patterns and the roles of clinical risk factors in geriatric trauma patients due to GLF or less.

## Material and Methods

### Study design

A retrospective observational study was conducted in the ED of an urban level I trauma center. Single center trauma registry data was used for study analysis. In this analysis, C-spine fracture trauma patients were evaluated and sub-divided into geriatric versus non-geriatric groups. Analysis was focused on geriatric trauma patients. The injury mechanism(s), location of C-spine injury, and potential clinical risk factors that could lead to associated injuries were all analyzed. This study was approved by the local institutional review board.

### Selection of participants

From January 1, 2006 till December 31, 2010, 12,805 patients were enrolled in this local trauma registry in which 726 patients had sustained cervical spine fracture(s) (Table 1). All trauma patients with C-spine fracture(s) were included in this study with focused analysis on geriatrics (age ≥ 65).

**Table 1:** Clinical Characteristics of Trauma Patients With Cervical Spine Fractures

	Geriatric Trauma Patients n = 139	Non-geriatric Trauma Patients n = 587	P
Age (Mean ± SD)	78.02 ± 8.52	37.15 ± 13.69	< 0.001
	(95%CI 76.59 - 79.45)	(95%CI 36.04 - 38.26)	
Gender (% Male)	56.83% (79/139)	74.11% (435/587)	< 0.001
Mechanism			
MVC	40.29% (56/139)	79.56% (467/587)	
Fall	53.96% (75/139)	12.44% (73/587)	
Assault	1.44% (2/139)	2.39% (14/587)	
Others*	4.32%(6/139)	5.62% (33/139)	< 0.001

SD: standard deviation; MVC: motor vehicle collision; * Others: including E-code 805.2 (pedestrian hit by rolling stock), 807.8 (railway accident of unspecified nature injuring other specified person), 826.1 (pedal cycle accident), 910.9 (accidental drown/submersion), 916.0 (struck accident by falling objects), 918.0 (caught accidentally in or between objects), 919.2 (machinery accident), 925.2 (accident electric current - industrial wires/appliance/machinery), and 958.8 (suicidal/self injury-hanging), etc. Basic characteristics of trauma patients with C-spine fractures. Geriatric trauma patients tended to have male in predominance (P < 0.001), sustain more C-spine injuries due to fall, whereas in non-geriatric patients C-spine injuries mainly occurred from MVC (P < 0.001).

### Study protocol

In this study, we defined patients older than 65 (including the age of 65) were geriatric patients. All C-spine fractures were recorded from trauma registry by evaluating the International Classification of Diseases (ICD-9) codes. The external causes of injuries were identified by E-code.

The type of C-spine fractures, its mechanism(s), and basic clinical characteristics were initially analyzed between geriatric and non-geriatric trauma patients (Table 2). Thereafter, Clinical variables from trauma patients due to GLF or less were intensely analyzed including age, gender, blood alcohol level (BAL), and associated injuries. As of associated injuries, facial laceration/abrasion, facial fractures, skull fractures, femur fractures, rib fractures, clavicle fractures, other extremity fractures excluding femur fractures, and intracranial pathologies (ICP) were included (Table 3). ICP was referred to any hemorrhagic intracranial lesions including intracranial contusion, subdural (SDH), epidural (EDH), subarachnoid hemorrhage (SAH), and intra-ventricular hemorrhage excluding isolated skull fractures.

**Table 2:** Type and Distribution of C-Spine Fractures in Trauma Patients

Location of C-spine Fractures	Trauma Patients with C-spine Fracture			Trauma Patient with C-spine Fracture due to GLF		
	Geriatric Trauma Patients	Non-geriatric Trauma Patients	P	Geriatric Trauma Patients	Non-geriatric Trauma Patients	P
	n = 139	n = 587		n = 35	n = 18	
C1	38 (27.34%)	80 (13.63%)	< 0.001	16 (45.71%)	5 (27.78%)	0.206
C2	75 (53.96%)	129 (21.98%)	< 0.001	20 (57.14%)	3 (16.67%)	0.005
C3	11 (7.91%)	47 (8.01%)	0.971	4 (11.43%)	3 (16.67%)	0.594
C4	18 (12.95%)	78 (13.29%)	0.916	6 (17.14%)	6 (33.33%)	0.182
C5	15 (10.79%)	110 (18.74%)	0.026	4 (11.43%)	5 (27.78%)	0.133
C6	19 (13.67%)	188 (32.03%)	< 0.001	2 (5.71%)	2 (11.11%)	0.481
C7	26 (18.71%)	243 (41.40%)	< 0.001	2 (5.71%)	0 (0%)	0.301
Single C-spine fracture	94 (67.63%)	378 (64.40%)	0.473	19 (54.29%)	15 (83.33%)	0.037

Different type and distribution of C-spine fractures in trauma patients. Among all trauma patients with C-spine fractures, geriatric patients tended to sustain more C1 and C2 fractures than non-geriatric patients (P < 0.001), whereas non-geriatric patients tended to sustain more C6 and C7 fractures (P < 0.001). Similar results showed on patients with C-spine fracture due to GLF with more non-geriatric patients sustained single C-spine fracture.

**Table 3:** Associated Injuries With C-Spine Fractures in Trauma Patients due to GLF or Less

	Geriatric Patients	Non-geriatric Patients	P
	(n = 35)	(n = 18)	
Gender (Male)	13 (37.14%)	14 (77.78%)	0.005
Upper C-spine fractures	26 (74.29%)	7 (38.89%)	0.012
ICP	7 (20%)	0	0.042
Clavicle fractures	0	1 (5.56%)	0.159
Facial/skull fractures	3 (8.57%)	4 (22.22%)	0.165
GCS (mean ± SD, 95%CI)	14.4 ± 1.8 (13.8 - 15.1)	14 ± 2.2 (12.8 - 15.1)	0.450
Rib fractures	1 (2.86%)	0	0.469
Hip/femur fractures	2 (5.71%)	2 (11.11%)	0.481
Facial laceration/abrasion	14 (40%)	7 (38.89%)	0.938

ICP: intracranial pathology; GCS: Glasgow Coma Scale; GLF: ground level fall; SD: standard deviation; CI: confidence interval; Upper C-spine fractures: C1, C2 spine fractures. The associated injuries with C-spine fractures in trauma patients due to GLF. Geriatric patients with C-spine fractures tended to occur more in female, sustained more fractures in upper C-spine, and co-existed more with ICP than non-geriatric patients (P < 0.05). The occurrence of other injuries showed no significant difference (P > 0.05).

E-codes were used to search for any trauma patients with GLF or less including: E880.1 (fall from tripping over a curb), E884.2 (fall from chair), E884.3 (fall from wheelchair), E884.4 (fall from bed), E884.6 (fall from commode, toilet), E885.0 (fall from non-motorized scooter), E885.1 (fall off roller skates, ice skates, in-line skates), E885.2 (fall from skateboard), E885.3 (fall while skiing), E885.4 (fall while snowboarding), E885.9 (GLF from slipping, tripping and then falling), E888.0 (GLF in which a sharp object is struck en route to ground), E888.1 (GLF in which a blunt object is struck en route to ground), and E888.8 (general GLF without other specifics). However, due to unable to determine the height from fall accurately, E881.0 (fall from ladder), E881.1 (fall from scaffolding), E882 (fall from or out of building or other structures), and E886.0 (collision or other cause of a fall during sports activities) were excluded from GLF in this study.

### Data analysis

Student t test was used to compare the continuous data and Chi square test was used to compare the categorical data between geriatric and non-geriatric groups. To compare the different BAL in geriatric versus non-geriatric groups, a one way analysis of variance (ANOVA) using the Bonferroni test was performed. In order to avoid confounding factors, multiple potential independent clinical variables were analyzed using multivariate logistic regression. All statistical analyses utilized Stata 12.0 statistical software (College Station, TX). A P value less than 0.05 was considered to be statistically significance.

An internal validation of random bootstrapping technique was applied to trauma patients sustaining C-spine fractures due to GLF by using Stata 12.0 (College Station, TX). When bootstrap was applied, it can randomly take certain amount of samples from the original data multiple times and generate a larger database from the study population sample [15]. Therefore, it is considered an internal validation technique. In this study, the data were bootstrapped 50 times and a final model was created. This model was then used for multivariate logistic regression analysis.

## Results

### Characteristics of study subjects

**Figure 1:**
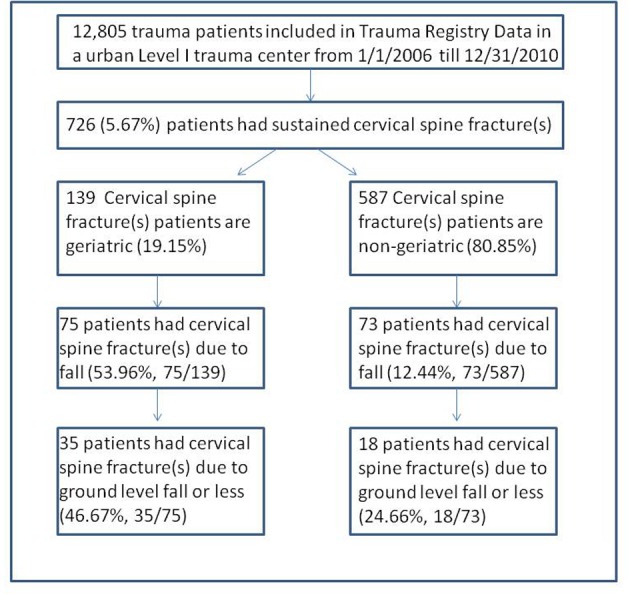
Flow diagram of trauma patients with cervical spine fractures. Among all trauma patients, 5.67% (726/12,805) patients had sustained C-spine fracture(s) and 19.15% (139/726) of C-spine fracture patients were geriatric. Furthermore, approximately half of these geriatric patients obtained C-spine fractures due to fall.

A total of 12,805 trauma patients were enrolled in the past 5 years (Jan. 2006 till Dec. 2010). Among all trauma patients, 5.67% (726/12,805) patients had sustained C-spine fracture(s) and 19.15% (139/726) of C-spine fracture patients were geriatric (Fig. 1). The mechanism(s) of C-spine injuries were different between geriatric and non-geriatric trauma patients. More geriatric patients had sustained C-spine fractures due to fall (53.96%), whereas, in non-geriatric group, motor vehicle collision (MVC) was the main cause of C-spine fractures (79.56%, P < 0.001) (Table 1).

Different locations of C-spine fracture between geriatric and non-geriatric trauma patients, between geriatric and non-geriatric trauma patients, different locations of C-spine fractures were analyzed. The results showed geriatric patients tended to sustain more upper C-spine (C1 and C2) fractures (P < 0.001) and non-geriatric patients had sustained more lower C-spine fractures (C6 and C7, P < 0.001). Similar injury pattern was found between geriatric and non-geriatric trauma patients due to GLF or less. Geriatric patients had sustained more upper C-spine fractures than non-geriatric group (C1: 45.71% vs 27.78% P = 0.206, C2: 57.14% vs 16.67% P = 0.005) (Table 2) but no difference was found in lower C-spine fractures. Furthermore, single C-spine fracture was found more in non-geriatric group (83.33% in non-geriatric group vs 54.29% in geriatric group, P = 0.037). Results from this study indicated that different C-spine injury pattern needs to be considered in geriatric trauma patients.

### ICP could be occurred with C-spine fractures in geriatric trauma patients due to GLF or less

C-spine fractures and intracranial pathology can result in both geriatric and non-geriatric trauma patients who sustain a GLF. However, geriatric patients tended to sustain more C-spine fractures and ICP than non-geriatric ones (Table 4) (P < 0.001). Moreover, associated injuries with C-spine fractures in trauma patients due to GLF or less were showed in Table 3 with ICP, facial laceration/abrasion, facial fractures/skull fractures, and femur fractures predominated. ICP associated with C-spine fractures were only found in geriatric patients in this study. Seven different clinical variables could potentially be independent risk factors associated with C-spine fractures and ICP in trauma patients due to GLF or less. The results of our multivariate regression showed only age (OR 1.17) and male gender (OR 91.57) were two independent risk factors (Table 5). As of relatively small sample size in trauma patients sustaining C-spine fracture due to GLF, random bootstrap technique was applied to expand the same size and logistic regression analysis was then validated internally. It showed similar results (age: OR 1.17, P = 0.042, 95%CI 1.00 - 1.36; male gender: OR 91.57, P = 0.002, 95%CI 5.52 - 1,516.40). Table 6 also listed all 7 geriatric C-spine patients with ICP, their mean ages were 81.85 ± 9.33 and 85.7% (6/7) were male.

**Table 4:** Intracranial Pathology (ICP) and C-Spine Fractures in Trauma Patients due to GLF

	Geriatric Trauma Patients due to GLF (n = 504)	Non-geriatric Trauma Patients due to GLF (n = 1,128)	P
C-spine fractures	35 (6.94%)	18 (1.59%)	< 0.001
ICP	32 (6.34%)	36 (3.19%)	0.0049
C-spine fracture and ICP	7 (1.38%)	0 (0%)	< 0.001

The occurrence of ICP and C-spine fracture in trauma patients due to GLF. It indicated that geriatric trauma patients tended to sustain not only C-spine fractures but also ICP as well. Additionally, only ICP and C-spine fracture co-existed in geriatric trauma patients due to GLF in this study (P < 0.001).

**Table 5:** Logistic Regression Analysis of Risk Factors of Head Injury in Trauma Patients With C-Spine Fractures due to GLF

Risk Factors	Adjusted Odds Ratio	P	95% Confidence Interval
Upper C-spine fractures	0.04	0.076	0.00 - 1.39
GCS	0.73	0.360	0.38 - 1.41
Age	1.17	0.031	1.01 - 1.35
Gender (male)	91.57	0.038	1.29 - 6,472.43
Facial Laceration/abrasion	0.10	0.219	0.00 - 3.90
Facial Fractures	0.45	0.639	0.01 - 12.29
Femur Fractures	3.17	0.504	0.10 - 94.40

Upper C-spine fractures including C1 and C2 fractures, GCS: Glasgow Coma Scale, Facial fractures including skull fractures. The results of logistic regression analysis. Potential risk factors that could predict the co-injury patterns of C-spine fracture and intracranial pathology in trauma patients were analyzed and adjusted odds ratios were showed. Two clinical variables (age and male gender) were considered independent risk factors to predict this co-injury pattern in trauma patients.

**Table 6:** List of C-Spine Trauma Patients With Intracranial Pathology due to GLF

	Age	Gender	ICP	C-spine Fractures	GCS	Other injuries
1	65	Male	SDH, SAH	C5	14	N/A
2	79	Male	SDH, SAH	C2	14	Femur Fracture
3	93	Male	EDH	C1	15	N/A
4	86	Male	SDH	C3	15	Facial laceration/abrasion
5	76	Male	SDH	C7	15	Facial Fractures
6	88	Female	SDH	C2	14	Facial laceration/abrasion
7	86	Male	SAH	C4	14	N/A

ICP: intracranial pathology; SDH: subdural hemorrhage; SAH: subarachnoid hemorrhage; EDH: epidural hemorrhage; GCS: Glasgow coma scale. The list of all C-spine fracture patients with associated ICP. All patients sustained C-spine fractures due to GLF. Their mean ages were 81.85 ± 9.33 and 85.7% (6/7) were male. None of these patients had GCS < 14.

### Alcohol played no roles in geriatric trauma patients with C-spine fractures due to GLF or less

The association between C-spine fractures and BAL was also analyzed in this study. BAL less than 80 mg/dL was considered non-toxic, BAL between 80 mg/dL and 200 mg/dL (not including 200 mg/dL) was defined as toxic, and more than 200 mg/dL was considered highly toxic. In our study, BAL was measured in 90.56% (48/53) of C-spine fracture patients due to GLF or less. Only one patient (3.3%, 1/30) reached toxic BAL in geriatric group. However, over 50% (9/17) of C-spine fracture patients had toxic BAL in non-geriatric group and the majority of these patients were highly toxic (77.8%, 7/9, P < 0.001) (Table 7). Our results showed alcohol had no roles to the cause of GLF or less in geriatric trauma patient with C-spine fractures due to GLF. We then performed secondary analyses involving all 53 patients to determine the potential effect on these missing BAL cases. When BAL was assumed to be toxic for all geriatric patients and non-toxic for all non-geriatric patients, we were still able to find statistically significant difference between geriatric versus non-geriatric patients (16.6% vs 50%, P < 0.001). When BAL was assumed to be highly toxic for all geriatric patients and non-toxic for all non-geriatric patients, results are very similar yet lower statistical significance reached (P = 0.020).

**Table 7:** The Role of Alcohol in C-Spine Fracture Trauma Patients due to GLF

Blood Alcohol Level (mg/dL)	Geriatric Trauma Patient	Non-geriatric Trauma Patient
	n = 31	n = 17
< 80 mg/dL	30	8
80 - 200 mg/dL*	1	2
≥ 200 mg/dL	0	7

*Not including 200 mg/dL. P < 0.001. The results of blood alcohol level (BAL) in trauma patients due to GLF. Only 48 patients had BAL recorded in trauma registry data. From these 48 patients, only one patient from geriatric group had toxic BAL, whereas over 50% of non-geriatric trauma patients had elevated BAL (P < 0.001).

## Discussion

In our study, geriatric trauma patients with C-spine fractures are considered special population. The majority of these patients had sustained C-spine fractures due to fall, whereas did not show the typical male predominance seen in non-geriatric patients. In geriatric trauma patients, falls were a much more common etiology and upper C-spine fractures predominated. Since basic characteristics showed significant different between geriatric versus non-geriatric patients, thus geriatric trauma patients should be considered to analyze separately from general trauma population.

Further analysis of trauma registry data showed that among all C-spine fracture patients due to fall, over 50% of geriatric trauma patients had sustained C-spine injuries due to GLF or less. In general, GLF is considered low energy mechanism with no significant traumatic injury. However, geriatric patients are less able to withstand the mechanical forces of trauma. In addition with the presence of comorbidities including osteoporosis, osteopenia, degenerated osseous changes could synergistically impact the severity of injury pattern even with minor trauma. Our results are in agreement with previous studies that geriatric patients tended to sustained more C-spine injuries than non-geriatric population due to GLF [16, 17].

Previous studies also found that geriatric patients tended to have more upper C-spine fractures whereas non-geriatric patients tended to have lower C-spine fractures [18]. This could partially due to the changes of cervical spine mobility. With increased degenerative changes in geriatric patients, lower C-spine segments (C4-C7), which are initially the most mobile motion segments of cervical spine become more stiffen. Therefore, C1-C2 motion segment become the most mobile portion [19]. Among all C-spine fracture patients, our results showed the same injury pattern with more geriatric patients sustaining C2 fractures due to GLF or less.

Geriatric trauma patients with C-spine fractures due to GLF can also sustain other associated injuries with intracranial pathology (ICP) and hip/femur fractures the most common ones [20]. Isolated ICP without skull fracture or facial fractures can be very subtle and clinically asymptomatic initially [21]. The significant biomechanical changes in the elderly could also make the results of physical examination unreliable [22]. Therefore, potential worsening clinical outcomes could be occurred later if underestimated. However, it is still uncertain under which circumstance could geriatric trauma patients be considered high risk to sustain associated injuries due to GLF. Some studies recommended CT of head and C-spine on every geriatric patient regardless of the mechanism since geriatric was considered one of the independent risk factors of predicting high occurrence of ICP associated with C-spine fractures [23, 24]. Other studies considered validated value of NEXUS study and recommended that NEXUS criteria was still able to apply to all trauma patients regardless of the advanced age and relatively unreliable physical examinations [25, 26]. Our study analyzed clinical variables that could be potential risk factors of predicting C-spine fractures with ICP in geriatric patients due to GLF. The results of study showed only male gender and age were two independent risk factors that could predict the associated ICP with C-spine fractures in geriatric patients. This was also validated internally. Whereas, other risk factors including femur fractures, facial fractures, skull fractures, facial laceration or abrasion were considered confounding factors. In addition, as of all 7 patients that had sustained C-spine fractures with ICP, their mean age was 81 years old which is also in agreement of the results of previous studies that focused on “old elder” trauma patients [27, 28]. In the contrary, none of the non-geriatric patients had sustained associated ICP with C-spine fractures due to low energy mechanism. These findings indicated the necessity of extensive image on geriatric trauma patients with minor injury.

Alcohol is considered one of the most common causes of trauma from GLF [29, 30]. The results of our study showed that over 50% of non-geriatric trauma patients due to GLF had toxic blood alcohol level (BAL). Furthermore, the BAL reached to highly toxic level (> 200 mg/dL) in majority of these patients. However, in geriatric trauma patients due to GLF, only one patient had positive BAL. It seemed that alcohol played no role in geriatric trauma patients due to GLF. Given the fact of less major traumatic injuries occurred in non-geriatric patients, it is hard to explain why certain number of patients had still sustained C-spine fractures. It is assumed that comorbidities could be existed to affect the normal bone structure of C-spine. One of the common causes of osteoporosis or osteopenia is alcoholism [31, 32]. Based on the animal study, alcohol affects the bone marrow stem cell differentiation resulting in decreased trabecular bone volume and significant cortical bone mineral density reductions [33, 34]. However, we are unable to know whether these non-geriatric patients were alcoholism or just occasionally consumed alcohol and whether other comorbidities that could affect the C-spine status in nontoxic ones. Further research needs to be designed to determine the alcoholic status and other comorbidities affecting non-geriatric trauma patient with C-spine fractures.

### Limitations

This was a retrospective study using the local trauma registry data from single urban level one trauma center. The retrospective methodology limits its applicability including bias regarding the accuracy of information, potential selection bias due to one institutional database, lack of follow up data, and missing data for analysis. We have limited missing data on BAL in trauma patients. By assuming all geriatric trauma patients being toxic and all non-geriatric patients being non-toxic allowed us to analyze BAL data and estimated two extremities. However, it is relatively common to analyze BAL and serum/urine drug test for substance abuse together. Due to not enough data about the status of substance abuse in these trauma patients, we were unable to analyze the status of substance abuse in this study. In addition, other clinical variables that could potentially associated with ICP and C-spine fractures were not included in this study, such as other extremity fractures, history of spine surgery, history of hypertension, diabetes, etc. With limited data in this study, bootstrap was also used to validate logistic regression analysis internally. However, this may cause skewed data and lead to unreliable analysis. Furthermore, due to the retrospective study and registry data with lack of follow up information, we were unable to determine the long term clinical outcomes in these patients. Overall, a large prospective study is required to externally validate the status of C-spine fractures due to minor trauma and further determine the independent risk factors of associated ICP with C-spine fractures in geriatric trauma patients.

### Conclusion

Overall, geriatric patients tend to sustain more upper C-spine fractures than non-geriatric patients regardless of the mechanisms. GLF or less not only can cause isolated C-spines fracture(s) but also lead to other significant injuries with intracranial pathological lesions as the most common one in geriatric patients. Advanced age and male are two risk factors that can predict this co-injury pattern. In addition, it seems that alcohol plays no role in the cause of ground level fall in geriatric trauma patients.

## Declaration

All authors have participated sufficiently in the study design, data collection, data analysis, and writing manuscript. Each author has reviewed the final version of the manuscript and approves it for publication. In addition, the authors declare no conflict of interests related to this manuscript.
